# Case Report: Challenges of Diagnosing Malaria in Returning Travelers at the Height of COVID-19 Pandemic

**DOI:** 10.4269/ajtmh.23-0467

**Published:** 2023-11-27

**Authors:** Edwin Kamau, Christian Olivo Freites, Ashlyn N. Sakona

**Affiliations:** ^1^ Department of Pathology and Laboratory Medicine, David Geffen School of Medicine, University of California, Los Angeles, California;; ^2^ Department of Pathology and Area Laboratory Services, Tripler Army Medical Center, Honolulu, Hawaii;; ^3^ Division of Infectious Diseases, David Geffen School of Medicine, University of California, Los Angeles, California

## Abstract

In 2021, we treated three patients in Southern California who contracted malaria while traveling in Uganda. Two patients visited the Nile River in Uganda in the months of July and August 2021, and upon returning to the United States, diagnosis was delayed due to limited access to care during the COVID-19 pandemic. One of the patients developed severe malaria, and the second developed parasitemia after he stopped taking malaria prophylaxis. The third patient, who traveled to Kampala, Uganda, in December 2021 returned home and was admitted for chronic medical conditions. Later in the clinical course, he developed symptoms consistent with malaria, but due to SARS-CoV-2 diagnosis, there was no suspicion of malaria infection until it was incidentally discovered while performing a blood manual differential. All patients were treated for malaria and recovered uneventfully.

## INTRODUCTION

In sub-Saharan Africa, malaria infections are on the rise after a downward trend,^1^ increasing travelers’ risk of acquiring an infection. Malaria should be high in the differential diagnosis in returned travelers with fever who may have visited malarious regions. The risk of developing severe malaria depends on the speed of diagnosis and treatment, as well as patient’s immune status.^2^ Residents of endemic areas develop premunition through repeated exposure, providing protection from illness but not from infection.^3^ It is unclear how long premunition persists after emigrating from endemic regions and whether individuals eventually revert to total malaria naivety.^3^^–^^5^ We present three malaria cases in returning travelers from Uganda in 2021, one of them a diasporan who emigrated from Rwanda, a malaria-endemic country, to the United States. The clinical presentations differed, demonstrating clinical diversity and diagnostic challenges of malaria infection and disease in returning travelers. In these patients, malaria diagnosis was delayed due to changes in medical care delivery during the COVID-19 pandemic.

### Case 1.

A 40-year-old male U.S. citizen returned after living and working in Uganda for 18 months. He lived in Kampala, where he worked from home. Before returning to the United States in mid-August 2021, he visited the Nile River, where he slept in a tent. Of note, he was not on any malaria prophylaxis during his 18-month stay in Uganda. After returning to the United States, 10 days after he went camping, he developed fevers and night sweats. He took over-the-counter acetaminophen and ibuprofen but symptoms persisted. He could not recall any particular pattern or time of the day that the fevers occurred. Due to restrictions and challenges of getting an appointment with providers at the height of the COVID-19 lockdown, he was only able to obtain a telemedicine consult 2 days after symptom onset. He was instructed to do a SARS-CoV-2 test, which was negative. He was prescribed oseltamivir, an antiviral drug that may reduce flu symptoms. However, his symptoms persisted, and on the 3rd day after the telemedicine consult, he presented to our emergency department (ED) with fevers, dry cough, nausea, diarrhea, and headache. Laboratory results showed thrombocytopenia, metabolic acidosis, hyperglycemia, hyperbilirubinemia, and elevated aspartate aminotransferase and alanine transaminase (ALT). Given the patient’s travel history and symptoms on presentation with a negative COVID-19 test, malaria was in the differential diagnosis. Malaria test was ordered and per the University of California Los Angeles’s clinical laboratory protocol, malaria rapid diagnostic test (mRDT; BinaxNOW™, Abbott, IL) and blood smears (thick and thin) were used to confirm malaria diagnosis. The mRDT was performed following manufacturer’s recommendations. Smears were prepared and read using standard methods following CDC recommendations and as previously published.^6^ Briefly, thick smears were prepared by placing and spreading a small drop of blood in the center of a clean, labeled slide and left to air dry for a minimum of 1 hour. Freshly prepared Giemsa stain was used for staining the slides. Thin smears were fixed in absolute methanol before staining. Slides were read (a minimum of 300 fields) by two senior technologists and confirmed by a pathologist or medical and public health laboratory microbiology fellow. Percent parasitemia was estimated by counting the number of infected red blood cells from the 1,000 cells counted.

**Figure 1.**
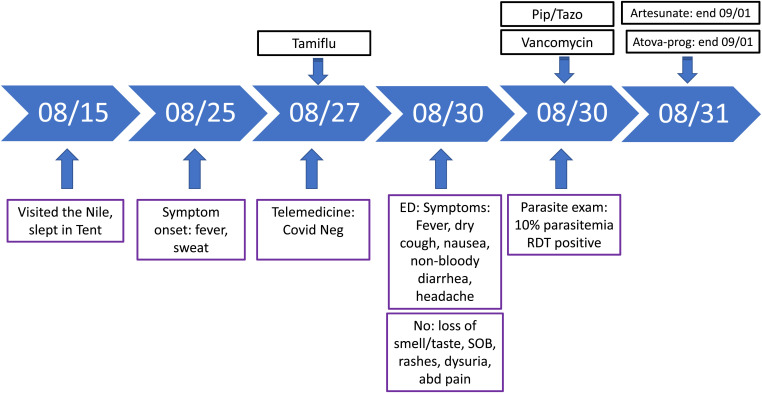
Patient 1 clinical course. abd = abdominal; ED = emergency department; Pip/Tazo = piperacillin/tazobactam; RDT = rapid diagnostic test; SOB = shortness of breath.

The patient had positive mRDT, and smears revealed 10% parasitemia of *Plasmodium falciparum*. Clinical manifestation of severe malaria per the CDC may include severe anemia (hemoglobin < 7 g/dL), acidosis, and/or percent parasitemia of ≥ 5%. Some remarkable laboratory test results from our patient included severe anemia (hemoglobin 3.14 g/dL; normal range 13.5–17.1), metabolic acidosis (lactate 61 mmol/L; normal range < 20 mmol/L) and thrombocytopenia (platelet count of 38 × 10^3^/µL; normal range 150–450 × 10^3^/µL). On the basis of CDC criteria, the patient was diagnosed with severe malaria and received intravenous artesunate and atovaquone-proguanil and recovered uneventfully. Because of COVID-19 lockdown and restrictions in accessing care, it took the patient 5 days from symptom onset to receipt of malaria diagnosis and treatment, which likely led to the development of severe malaria (Figure 1).

### Case 2.

**Figure 2.**
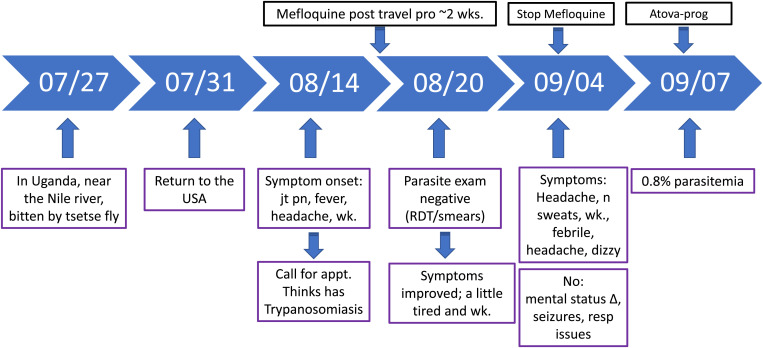
Patient 2 clinical course. Δ = change; appt = appointment; Atova-prog = Atovaquone/Proguanil; jt pn = joint pain; resp = respiratory; wk = weak.

A 57-year-old male U.S. citizen who is an exotic animal veterinarian returned from Uganda 3 days after he noted having been bitten by a tsetse fly near the Nile River in July 2021. Figure 2 provides the patient’s travel history and follow-on disease events. While in Uganda, he was continuously on mefloquine prophylaxis. Two weeks after returning to the United States (while still on mefloquine), he developed joint pain, fever, headache, and weakness. He called his provider and requested an appointment, but, even in light of his acute symptoms, because of the COVID-19 lockdown and the related difficulty in getting appointments, it took 6 days to be seen by a doctor. He was convinced that he had African trypanosomiasis from the tsetse fly bite, explaining his insistence on seeing the doctor and not visiting urgent care or telemedicine services. Six days after symptoms onset, and now ∼3 weeks after returning from his trip, microscopic examination of Giemsa-stained blood smears and mRDT performed in our center were negative for malaria. Further, there was no evidence of any other parasite in the blood smears, including trypanosomes. The patient’s symptoms improved, and after a negative malaria test, he stopped taking mefloquine prematurely. Two weeks after the first doctor’s visit (now 5 weeks after returning), he developed a recurrence of fevers, with headaches, night sweats, weakness, and dizziness. Repeated laboratory testing for malaria now showed 0.8% parasitemia with *P. falciparum* and positive mRDT, as well as elevated ALT. He was treated with atovaquone-proguanil and recovered uneventfully.

### Case 3.

**Figure 3.**
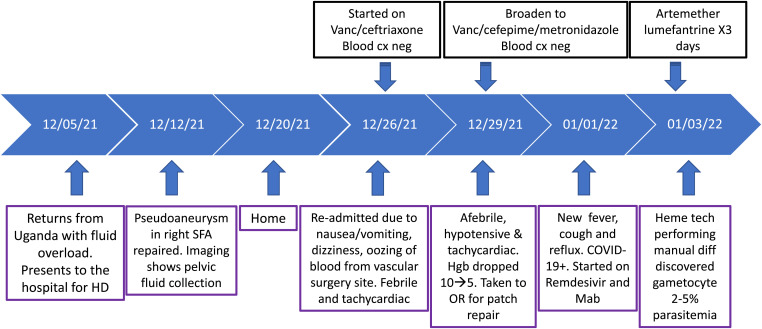
Patient 3 clinical course. cx = culture; HD = hemodialysis; Mab = monoclonal antibodies; OR = operating room; SFA = superficial femoral artery; Vanc = vancomycin.

A 74-year-old male with diabetes mellitus type 2, hypertension, and end-stage renal disease was admitted for hemodialysis immediately after he returned from a 3-week trip to Uganda in December 2021. Originally from Rwanda, he lived in California at the time of presentation. Figure 3 provides the patient’s clinical events of 1 month’s duration after returning from travel. A week after admission, he underwent an uncomplicated repair of a pseudoaneurysm in the right superficial femoral artery (SFA). A week after the surgery, he was discharged home but was readmitted 1 week later with nausea/vomiting, dizziness, and blood oozing from the surgical site. He was febrile, tachycardic, and had elevated lipase and lactate.

**Figure 4.**
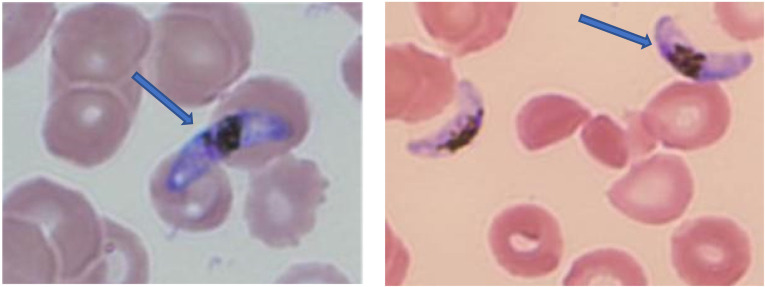
Pictures showing *Plasmodium falciparum* gametocytes discovered from blood manual differential of patient 3.

Abdominal tomography showed postsurgical changes around the SFA surgical site and surrounding skin and soft tissue edema. He was treated with antibiotics (Figure 3) for possible soft tissue infection and remained afebrile for the next 5 days. On day 3 after readmission, his hemoglobin dropped, and he was hypotensive and tachycardic. Due to the worsening of symptoms, his antibiotics were broadened to include metronidazole. On day 6, he developed a cough and fever. He tested positive for SARS-CoV-2 and was given remdesivir and sotrovimab. His symptoms did not improve. Two days later, now 30 days after returning from Uganda, a technologist performing a blood manual differential incidentally discovered *P. falciparum* gametocytes with estimated 2–5% parasitemia (Figure 4). It is plausible that the malaria diagnosis, which was not in the differential diagnosis, was delayed by more than a week due to confounding clinical events, including COVID-19 diagnosis. The symptoms that led to readmission (on day 21 after the initial admission) and subsequent clinical events, including fever, elevated lipase and lactate, decrease in hemoglobin, hypotension, tachycardia, and the presence of gametocytes, were consistent with malaria clinical manifestation. After the diagnosis, the patient was treated with artemether/lumefantrine and recovered uneventfully.

## DISCUSSION

Early initiation on treatment is important in reducing morbidity and mortality from malaria. The incubation period of *P. falciparum* is 7 to 14 days after an infectious mosquito bite, and mature gametocytes usually appear 7 to 15 days after the initial wave of asexual parasites.^7^^,^^8^ We present three travelers who returned from Uganda in 2021 and were diagnosed with malaria during the height of COVID-19 pandemic. Malaria diagnosis was delayed either due to difficulty in getting an appointment or coinfection with SARS-CoV-2 virus. The first patient described had severe malaria (parasitemia > 5%), likely due to delay (5 days) between onset of symptoms and presentation at our ED. When the patient first developed symptoms, he had a telemedicine consult, and although he was SARS-CoV-2 negative, he was prescribed oseltamivir, further delaying care; in light of his travel history, malaria should have been high on the differential diagnosis but was initially missed. The second patient, an exotic animal veterinarian who had traveled to the Nile River for work claimed to have been bitten by tsetse fly, and although still on malaria prophylaxis (mefloquine), he was more concerned about African trypanosomiasis infection. It took about a week from symptom onset to receipt of care, which may be linked to challenges of obtaining care during the pandemic. Given his travel history, malaria was in the differential diagnosis but was initially not detected, likely due to mefloquine prophylaxis. Mefloquine is a long-acting blood schizonticide but is not active against hepatic schizont replication and merozoite production. Malaria-naive patients can develop symptoms even with undetectable blood parasites,^9^ which may explain the patient’s initial symptoms when no parasite is detected. The negative blood results led the patient to stop the malaria prophylaxis prematurely, which led to detectable parasitemia 2 weeks later, within the terminal half-life of mefloquine of 14–15 days.^8^^,^^10^ In the third case, the patient’s health comorbidities, including coinfection with SARS-CoV-2, may have made malarial infection low in the differential diagnosis. In hindsight, it is evident that the symptoms that the patient presented with during readmission were consistent with malaria clinical manifestation. Further, being a diasporan who had emigrated from Rwanda to the United States, the patient’s immune system may have allowed him to tolerate the infection much more than the first two cases, who were presumably malaria-naive patients. This patient likely had circulating asexual parasites for some time, given the presence of mature gametocytes. Individuals with repeated previous exposure during their residency in endemic areas have larger magnitude and breath of detectable antibody responses toward *P. falciparum* exposure compared with malaria-naive individuals.^5^

## CONCLUSION

These cases demonstrate the challenges associated with diagnosing malaria in non–malaria-endemic regions brought on by the changes in the delivery of care during COVID-19 pandemic. Complications associated with malaria infection can be reduced with prompt diagnosis and treatment. It is therefore important that a high index of suspicion for malaria is maintained in all individuals with a travel history to malaria-endemic regions with a compatible syndrome, regardless of comorbid conditions, use of antimalarial prophylaxis, and co-infections.

## References

[1] WHO , 2021. World Malaria Report. Geneva, Switzerland: World Health Organization.

[2] Mascarello M Allegranzi B Angheben A Anselmi M Concia E Laganà S Manzoli L Marocco S Monteiro G Bisoffi Z, 2008. Imported malaria in adults and children: epidemiological and clinical characteristics of 380 consecutive cases observed in Verona, Italy. J Travel Med 15: 229–236. 18666922 10.1111/j.1708-8305.2008.00204.x

[3] Mischlinger J Rönnberg C Álvarez-Martínez MJ Bühler S Paul M Schlagenhauf P Petersen E Ramharter M, 2020. Imported malaria in countries where malaria is not endemic: a comparison of semi-immune and nonimmune travelers. Clin Microbiol Rev 33: e00104–e00119. 32161068 10.1128/CMR.00104-19 PMC7067581

[4] Mascarello M Gobbi F Angheben A Concia E Marocco S Anselmi M Monteiro G Rossanese A Bisoffi Z, 2009. Imported malaria in immigrants to Italy: a changing pattern observed in north eastern Italy. J Travel Med 16: 317–321. 19796101 10.1111/j.1708-8305.2009.00321.x

[5] Yman V White MT Asghar M Sundling C Sondén K Draper SJ Osier FHA Färnert A, 2019. Antibody responses to merozoite antigens after natural *Plasmodium falciparum* infection: kinetics and longevity in absence of re-exposure. BMC Med 17: 22. 30696449 10.1186/s12916-019-1255-3 PMC6352425

[6] Cohen R Feghali K Alemayehu S Komisar J Hang J Weina PJ Coggeshall P Kamau E Zapor M, 2013. Use of qPCR and genomic sequencing to diagnose *Plasmodium ovale* wallikeri malaria in a returned soldier in the setting of a negative rapid diagnostic assay. Am J Trop Med Hyg 89: 501–506. 23836567 10.4269/ajtmh.12-0724 PMC3771289

[7] Day KP Hayward RE Dyer M, 1998. The biology of *Plasmodium falciparum* transmission stages. Parasitology 116: S95–S109. 9695114 10.1017/s0031182000084985

[8] Andagalu B , 2023. Malaria transmission dynamics in high transmission setting of western Kenya and the inadequate treatment response to artemether-lumefantrine in an asymptomatic population. Clin Infect Dis 76: 704–712. 35767269 10.1093/cid/ciac527 PMC9938745

[9] Kamau E Bennett JW Yadava A, 2022. Safety and tolerability of mosquito bite-induced controlled human infection with *Plasmodium vivax* in malaria-naive study participants – clinical profile and utility of molecular diagnostic methods. J Infect Dis 225: 146–156. 34161579 10.1093/infdis/jiab332

[10] Gutman J Green M Durand S Rojas OV Ganguly B Quezada WM Utz GC Slutsker L Ruebush TK 2nd Bacon DJ, 2009. Mefloquine pharmacokinetics and mefloquine-artesunate effectiveness in Peruvian patients with uncomplicated *Plasmodium falciparum* malaria. Malar J 8: 58. 19358697 10.1186/1475-2875-8-58 PMC2674465

